# Review of Recent Advances in Predictive Maintenance and Cybersecurity for Solar Plants

**DOI:** 10.3390/s25010206

**Published:** 2025-01-02

**Authors:** Younes Ledmaoui, Adila El Maghraoui, Mohamed El Aroussi, Rachid Saadane

**Affiliations:** 1 Laboratory Engineering System, Hassania School of Public Works, Casablanca BP 8108, Morocco; mohamed.elaroussi4@gmail.com (M.E.A.); saadane@ehtp.ac.ma (R.S.); 2 Green Tech Institute, Mohammed VI Polytechnic University, Benguerir BP 43150, Morocco; adila.elmaghraoui@um6p.ma

**Keywords:** artificial intelligence, cybersecurity, predictive maintenance, renewable energy, solar plant, review

## Abstract

This paper presents a systematic review that explores the latest advancements in predictive maintenance methods and cybersecurity for solar panel systems, shedding light on the advantages and challenges of the most recent developments in predictive maintenance techniques for solar plants. Numerous important research studies, reviews, and empirical studies published between 2018 and 2023 are examined. These technologies help in detecting defects, degradation, and anomalies in solar panels by facilitating early intervention and reducing the probability of inverter failures. The analysis also emphasizes how challenging it is to adopt predictive maintenance in the renewable energy industry. Achieving a balance between model complexity and accuracy, dealing with system unpredictability, and adjusting to shifting environmental conditions are among the challenges. It also highlights the Internet of Things (IoT), machine learning (ML), and deep learning (DL), which are all incorporated into solar panel predictive maintenance. By enabling real-time monitoring, data analysis, and anomaly identification, these developments improve the accuracy and effectiveness of maintenance procedures.

## 1. Introduction

**Figure 1:**
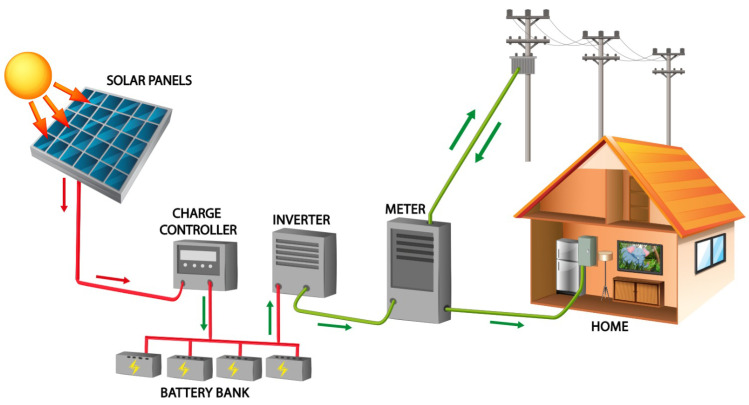
Components in a photovoltaic system.

The key technical tasks involved in photovoltaic plant maintenance are inspections, verifications, repairs, and replacements. To ensure that the factory operates without a hitch, these tasks are completed on a regular basis. Preventive maintenance is the term for this form of upkeep, and it adheres to the instructions given by the particular PV component makers. These rules specify the manner and frequency of verifications, as well as the time for replacement. With the advent of Maintenance 4.0, leveraging real-time data and predictive analytics can further optimize these tasks, enabling a shift from routine preventive maintenance to a more proactive and data-driven approach. However, even with these advancements, unanticipated failure situations may still necessitate urgent maintenance and repair operations to reduce income losses. All the procedures required to return the system to normal operation are included in what is referred to as corrective maintenance [1]. Almost all PV plants perform both preventive and corrective maintenance, and efforts to develop more effective and economical methods appear to be making little headway [2]. A predictive maintenance policy uses monitoring data [3,4] and machine learning algorithms [5,6] to foresee system faults and arrange maintenance interventions accordingly. In contrast to the planned nature of preventive and corrective maintenance [7], this approach involves continuous data collection through an array of sensors, providing real-time information about the plant’s operation in order to increase the lifecycle and reduce the maintenance costs and operational downtime of the components in a photovoltaic system (Figure 1).

### 1.1. Predictive Maintenance

**Figure 2:**
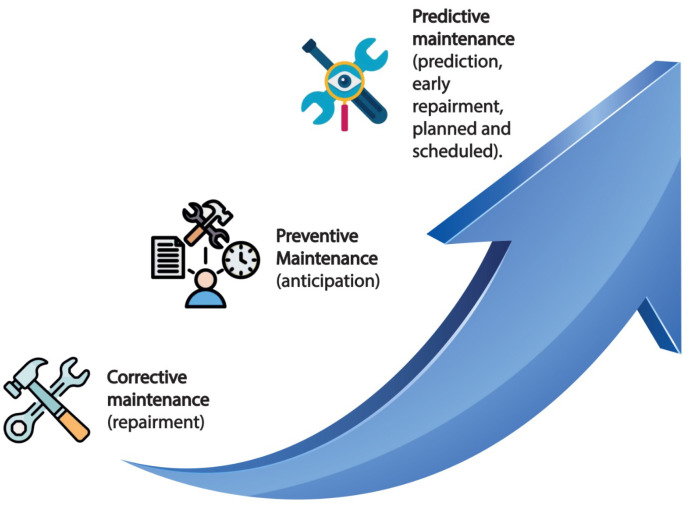
Maintenance types.

High levels of competence in data gathering, data analysis, and machine learning are required to create a predictive maintenance system. This competence requires the participation of specialists in solar technology who can offer insights into the technical facets of PV plant operation, seasoned data scientists who are in charge of model creation, and monitoring partners who can guarantee a steady flow of high-quality data. Predictive maintenance is a type of condition-based maintenance [8,9] that involves identifying typical trends in the deterioration of key plant component parameters. The condition of out-of-service equipment can be assessed using predictive techniques to determine when and whether maintenance is necessary. It serves several key objectives: minimizing downtime, extending equipment lifespan, reducing costs, enhancing energy efficiency, enhancing planning, and data-driven decision making. There are three basic types of maintenance in PV plants [10], as shown in Figure 2, each serving a distinct purpose, which can be explained as follows:

- **Preventive Maintenance:** Preventive maintenance is a scheduled and planned approach to maintenance. It involves regular technical inspections, repairs, and replacement activities that are carried out at predetermined intervals. It aims to maintain the PV plant’s regular operation and avoid potential problems.
- **Corrective Maintenance:** This type of maintenance is reactive in nature. When unexpected failure events happen in the PV plant, it comes into play. To prevent further revenue losses and return the system to normal operation, immediate maintenance and repair interventions are needed in these situations.
- **Predictive Maintenance:** A data-driven, forward-thinking approach to maintenance is predictive maintenance. It uses monitoring data and machine learning algorithms to foresee potential system failures rather than fixed schedules, as with preventive and corrective maintenance [11].

**Figure 3:**
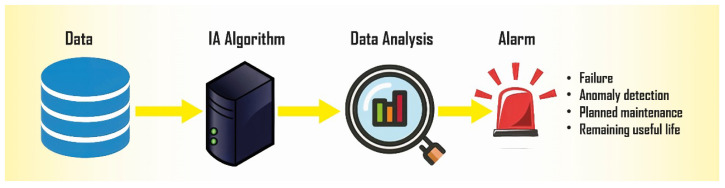
Predictive maintenance schema.

Predictive maintenance uses connected sensors to gather data from machinery and equipment and relies on cutting-edge technologies like the IoT [12] and machine learning [13]. Alerts are then sent to maintenance teams after these data are processed in real time to evaluate the state of the equipment and identify any potential flaws. Additionally, improvements in machine learning enable predictive maintenance to predict the condition of the equipment in the future, thereby optimizing maintenance workflows and enhancing efficiency. Gathering data makes these predictions more accurate, ensuring that equipment performs at its best. Figure 3 depicts the predictive maintenance schema. Advanced predictive maintenance strategies rely on the use of historical data to define a baseline model, which is then used to analyze new streaming data in order to identify anomalous behavior. The observed anomalies are then correlated with impending failures. Data analysis uses the results of feeding new data to the baseline model to detect sustained deviations in the behavior of the components. Additionally, appropriate filters are applied to the output data to reduce the number of false alarms. The baseline model represents the behavior over the range of the component’s operational conditions. The results of data analysis, along with data generated by other auxiliary techniques, can be further processed to determine the impact and potential causes of failure. Hence, we will emphasize both the aspect of monitoring solar energy production [14] and the aspect of its forecasting using machine learning algorithms [15,16] equally in this review.

### 1.2. Smart Grids and Cybersecurity Challenges

**Figure 4:**
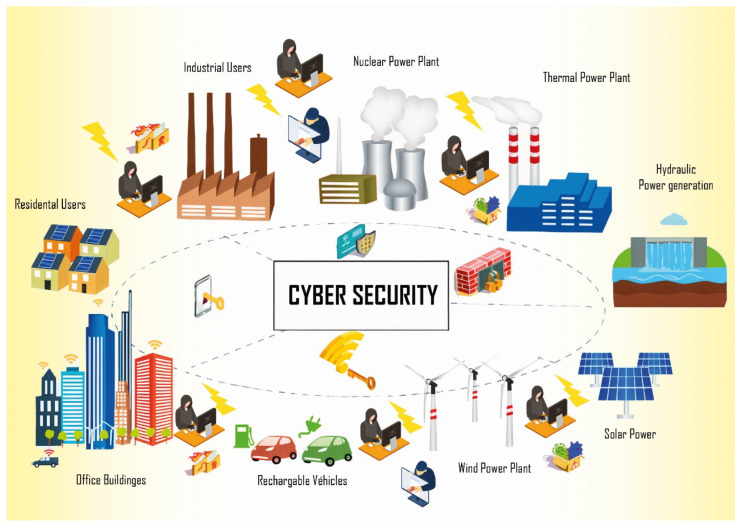
Cybersecurity in smart grids [19].

Smart grids rely on advanced communication and control technologies to manage energy distribution efficiently, making them vulnerable to both cyber and physical threats [17], as shown in Figure 4. To safeguard these systems, it is crucial to implement cybersecurity measures, ensuring the protection of both the solar components and the broader infrastructure. As the penetration of renewable energy sources, including solar power, increases within smart grids, the need for robust cybersecurity strategies becomes even more critical. The application of best practices for securing solar plants can provide valuable insights for enhancing the resilience and reliability of smart grids, protecting them against potential cyberattacks and disruptions [18]. Smart grids, which enable real-time monitoring and control of energy distribution, can benefit significantly from the cybersecurity measures outlined in this paper. The integration of solar energy sources within smart grids requires robust protection mechanisms to prevent cyber threats and ensure system reliability. The best practices discussed for securing solar plants can also be extended to these systems to protect the interconnected infrastructure.

### 1.3. Importance of Cybersecurity in Solar Plants

Cybersecurity for solar energy is mainly implemented to guarantee the security [20], safety, and reliability of solar energy along with the financial stability of solar power plants. Based on historical promises, cybersecurity requirements for solar plants were developed to prevent cyber incidents as much as possible. They not only comply with normative and regulatory frameworks but also align with the best security industry practices and standards at the international level [21]. In principle, a system that is safe by design, able to recover when necessary in safety mode, and guarantees the secure control of a controlled safe transition from the previous states is also a secure system. However, this may not be sufficient, especially in light of the use of attack techniques that could compromise operational capabilities, cause disruption, and eventually overwhelm defense systems [22].

### 1.4. Cybersecurity Threats in Solar Plants

**Figure 5:**
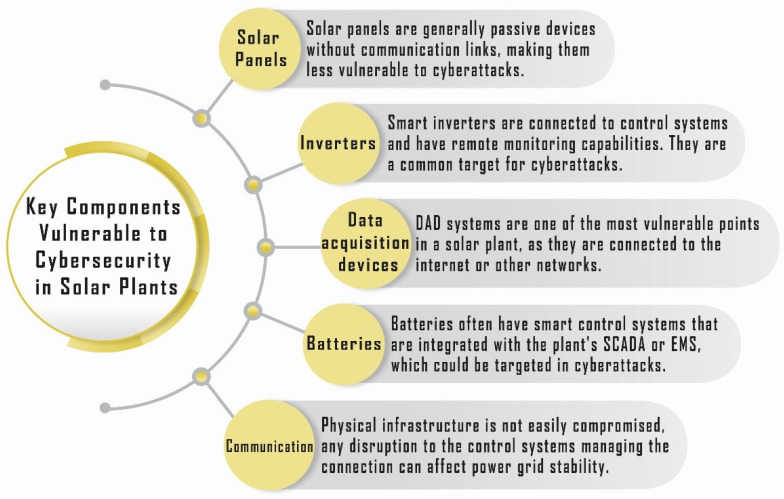
Cybersecurity in solar plants.

A solar power plant operates as an intricate system comprising multiple interdependent components, each critical to the seamless conversion of solar energy into usable electrical power. These components work in harmony to ensure optimal energy generation, storage, and distribution. Figure 5 provides an overview of the primary components in a solar power plant and highlights the elements that are particularly susceptible to cybersecurity threats [23]. Among the most vulnerable components are the following:

- **Inverters (Smart Inverters):** Responsible for converting a direct current (DC) produced by solar panels into an alternating current (AC) for grid compatibility, these devices increasingly incorporate smart functionalities, making them potential targets for cyberattacks [24].
- **Data Acquisition Devices:** Essential for monitoring and recording plant performance, these devices often serve as entry points for unauthorized access if not adequately secured.
- **Communication Networks:** The backbone of data transmission both within and beyond the plant, these networks facilitate remote monitoring and control but can be exploited to disrupt operations or extract sensitive information.
- **Battery Storage Systems:** Critical for storing surplus energy, these systems rely on advanced software for management, which can be compromised to interfere with energy availability or efficiency.

These vulnerabilities underscore the growing importance of robust cybersecurity measures to safeguard the integrity and reliability of solar power plants in an increasingly interconnected energy landscape.

### 1.5. Practical Cases and Applications

#### 1.5.1. Predictive Maintenance for PV Plants

Predictive maintenance in solar plants has been effectively demonstrated through the development of a hybrid AI model by researchers at ZHAW as part of the Innosuisse project [25]. The model integrates data-driven artificial intelligence with domain expertise (physics-informed AI) to optimize maintenance decisions. Deployed in collaboration with Fluence Energy, the system identifies energy loss events caused by issues such as soiling, electronic failures, or tracker errors and determines the optimal time for maintenance. By incorporating historical data and physical knowledge, the AI model creates realistic fault scenarios to train algorithms, enabling early diagnosis and cost-effective intervention. This approach minimizes energy losses, enhances system reliability, and supports the efficient operation of solar power plants. Moreover, the SmartHelio solution [26], implemented in Lausanne, Switzerland, leverages IoT hardware and machine learning (ML) frameworks to enhance fault detection capabilities in photovoltaic (PV) systems. This solution addresses the significant impact that faults in individual PV modules can have on the overall power efficiency, reliability, and safety of the system. Faults, if not detected promptly, can lead to safety risks and fire hazards. By integrating low-cost sensors that monitor electrical and thermal parameters at the module level, SmartHelio enables the real-time detection of faults. The system emulates eight common PV module faults and applies them to a real PV system, collecting and labeling panel-level data for analysis. Different ML models were used to classify these faults, with neural networks achieving 93% classification accuracy for seven fault types, showcasing the potential of advanced fault detection systems in improving PV system performance and safety.

#### 1.5.2. Cyberattacks in PV Plants

The first-ever cyberattack on Ukraine’s power grid demonstrated the devastating impact of attackers hacking critical cyber assets, specifically substation networks and local control centers [27]. Attackers executed disruptive switching operations across multiple substations using compromised supervisory control and data acquisition systems by gaining administrative privileges. This coordinated attack caused significant instability, potentially leading to cascading system-wide failures. Furthermore, in the USA, in May 2019, a cyberattack on wind and solar assets, particularly affecting solar systems, exposed significant vulnerabilities in the energy sector’s cybersecurity practices [28]. The attack, which exploited an unpatched Cisco firewall, caused a denial-of-service (DoS) disruption, affecting 500 MW of wind and solar capacity in California. The incident demonstrated how basic security lapses, such as failing to update firewalls, can put energy networks at risk. Experts view this as a wake-up call, emphasizing that utilities must improve security maintenance, including patching and monitoring network devices, to protect against future vulnerabilities.

The remainder of this paper is structured as follows. Related works are discussed in Section 2. The methodology is presented in Section 3. Section 4 focuses on the results and discussion. The conclusions based on the outcome of the analysis phase are presented in Section 5.

## 2. Related Works

Numerous studies have been undertaken on monitoring solar energy, and many successful implementations and approaches have demonstrated the benefit in sectors such as solar plant production. Forecasting IoT energy usage is a prominent topic, and research into it using machine learning and deep learning methods has progressed quickly. Ali Reza Sajun et al. [29] constructed an edge-based individualized anomaly detection in large-scale distributed solar farms, based on Siamese-twin neural networks. Suprita M. Patil et al. [30] investigated a system for the online display of the power usage of solar energy as renewable energy. This monitoring is achieved through a Raspberry Pi using the Flask framework. Ambuj Gupta et al. [31] proposed using a Raspberry Pi to monitor solar output in real time. Saravanan et al. [32] used a low range of sensors along with a stepper motor to define high throughput. Ledmaoui et al. [33] used a data logger based on an ESP32 card to monitor inverter output, and the data were saved in the Firebase database. Time-series forecasting research has become more complex. In one study, the researchers developed more sophisticated machine learning architectures, specifically designed for forecasting time-series data. Mokhtar Jlidi et al. [34] used the ANN algorithm for the purpose of predicting the PV system’s current and voltage by predicting the PV system’s operating temperature and radiation. Additionally, Ayadi Osama et al. [35] proposed an efficient artificial neural network (ANN) model in which internal parameters are formulated to effectively map the inputs to the outputs. In another study, Khalid Anwar and Sandip Deshmukh [36,37] found that the correlation coefficients between the ANN predictions and the actual mean monthly global solar radiation intensities for the training and testing datasets were higher than 95 percent. Furthermore, researchers have developed more advanced deep learning architectures specifically for forecasting solar plant fault detection. Zhendong Huang et al. [38] proposed an efficient method based on a convolutional neural network (CNN) to detect solar panel edges in infrared images. They used many images collected from different photovoltaic plants to verify the effectiveness of the proposed fault diagnosis algorithms. In another study, Tamilselvi Selvaraj  et al. [39] focused on finely tuning pre-trained models of convolutional neural networks, specifically AlexNet, GoogleNet, and SqueezeNet. Based on the performance metrics, SqueezeNet was used for training thermal images of solar panels and for the classification of environmental faults. The evaluation of various edge-based anomaly detection system implementations was described by the authors of [29] using the ANN algorithm. Furthermore, the authors of [40] focused on using an ANN and a type-2 fuzzy logic system to detect PV anomalies. The authors of [41] employed ML algorithms in the RapidMiner tool, using real-time data from a smart grid in an experimental open-pit mine. Four ML algorithms (artificial neural network (ANN), support vector machine (SVM), decision tree (DT), and random forest (RF)) were compared using metrics such as correlation, mean absolute error, root mean square error, and root relative squared error. The results showed that RF performed the best for energy forecasting in this context. The authors of [42] proposed various machine learning algorithms, including support vector machine (SVM), artificial neural network (ANN), decision tree (DT), and random forest (RF), to predict the electrical energy consumption of hotel buildings. A case study of a hotel in Shanghai was presented alongside a comparative study of these algorithms. The best-fitting algorithm was selected for further research and improvements, with potential applications in simulation software and by energy managers for cost estimation, power-shedding solutions, and integration of Distributed Energy Resources (DERs). In [43], the authors proposed a hybrid artificial intelligence framework for power transformers, incorporating various diagnostic algorithms, health index, and life-loss estimation methods to manage the challenges brought by the integration of distributed energy resources, energy storage systems, and electric vehicle charging stations in the grid. The solution includes a comparative study of different algorithms to select the most suitable models. The developed architecture interacts with machine learning models and is connected to an online monitoring system, which helps calculate the key performance indicators and manage alarms, load management, power factor control, and maintenance schedules. The authors of [44] focused on maintaining the power transformer, a crucial element in the electrical grid, using predictive maintenance techniques, such as thermal and vibration analysis, wavelet transform, and dissolved gas analysis. The application of these methods in the Smart Energy Management System can reduce failures and prevent blackouts. The paper highlighted different failure classification techniques using dissolved gas analysis data, including logistic regression, multiclass jungle trees, decision trees, and artificial neural networks. The authors demonstrated how to integrate these techniques into real-time energy management systems using sensors and offline database interactions to enhance monitoring and diagnostics in a micro-grid. The authors of [45] presented an artificial intelligence-based diagnostic technique for detecting broken rotor bars in induction motors, which are commonly used in electric traction systems. The research used datasets of healthy and malfunctioning motors, capturing transient current and voltage signals during motor start and steady state. The data were converted from the time domain to the frequency domain using the fast Fourier transform, preprocessed, and then used in a supervised machine learning model to evaluate the motor’s operation. The goal was to ensure motor integrity and availability in electric vehicles through onboard diagnostic and prognostic tools. The seamless integration of these functionalities within the edge devices enhances the efficiency, reliability, and performance of solar energy monitoring systems. It provides real-time insights and actionable information to stakeholders, enabling them to optimize energy utilization, reduce operational costs, and enhance system reliability. The authors of [46] discussed the rise of digital technologies like the Internet of Things, big data, cloud computing, artificial intelligence, and digital twins (DTs) in various sectors, including mining. The use of DTs offers improved efficiency, productivity, and sustainability, but integration into the mining business remains a challenge. The paper provided insight into the use of DTs through case studies and discussed a DT multi-layered architecture for the mining industry in the context of Industry 4.0 and value lifecycle management, aiming to inspire future research [47]. In this context, there has been little research on renewable energy, particularly in solar energy and more specifically in monitoring and forecasting with predictive maintenance. By combining real-time monitoring data with machine learning algorithms, we can achieve enhanced predictive capabilities. This synergy allows for the early identification of potential issues, facilitating proactive maintenance interventions. A relevant study on predictive maintenance for PV systems was conducted in [48]. The authors summarized approaches and opportunities for PdM conducted between 1999 and 2017, focusing on four approaches: manual diagnostics, failure modes and effects analysis (FMEA), machine learning, and real-time monitoring. The approach proposed in [49] is based on calculating the numerical difference between data from the real plant and data estimated using the ANN algorithm for power production based on the inverter model developed.

In this study, our primary objective is to enhance the effectiveness of predictive maintenance techniques for solar power plants by assimilating cutting-edge research findings. We aim to bolster existing methodologies by incorporating the latest advancements in thermal imaging, the Internet of Things (IoT), and machine learning. This study recognizes the pivotal role of thermal cameras [50] in detecting potential issues and further explores the integration of the IoT for real-time data acquisition and analysis. Machine learning algorithms play a central role in processing vast datasets, enabling more accurate predictions and proactive maintenance strategies [51,52,53]. The authors of [54] showed that while Smart Inverters (SIs) enhance grid support, they also introduce cybersecurity risks, particularly in distribution networks. The authors of [55] explored how IoT integration enhances efficiency and sustainability in renewable energy production while also exposing systems to cyberattacks, highlighting vulnerabilities, such as insecure protocols and poor encryption exploited by threats like false data injection and denial-of-service attacks.

We focus on innovative methods like drones to improve monitoring and inspections. The purpose is to determine how solar plants can operate more efficiently and become more reliable and sustainable by embracing the technological innovations of the past five years.

## 3. Methodology

### 3.1. Planning Protocol

The following review planning protocol was employed for this paper:

- Research questions:

Q1. What are the recent methods used to perform PdM in solar systems?
Q2. Which equipment is used with PdM techniques in solar systems?
Q3. What is the most effective way to carry out PdM in solar systems?

- Databases for literature review:

The well-known, scientifically focused literature databases, MDPI, IEEEXplore, ScienceDirect, and Elsevier, were the sources of papers for this systematic review.

- Exclusion criteria:

E1. Works not related to PdM in solar systems.
E2. Works with fewer than four citations.
E3. Works published before the year 2018.

- Quality criterion:

Qc1. Papers published after 2017 that are PdM-related and have more than three citations.

**Figure 6:**
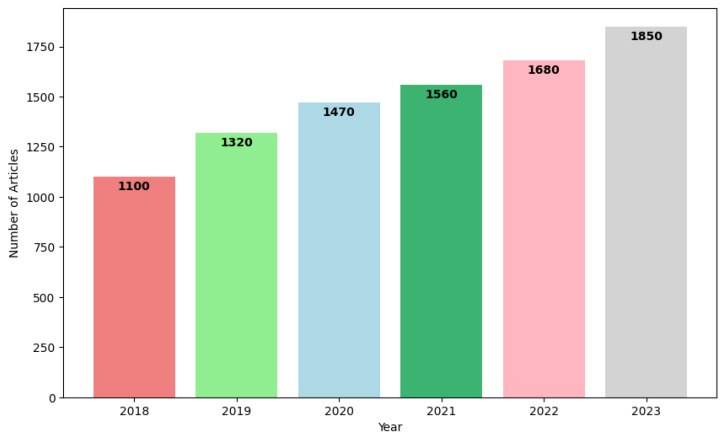
Evolution of articles on predictive maintenance and cybersecurity for solar plants published between 2018 and 2023.

- Execution:

Based on the exclusion criteria E1, E2, and E3, 20 out of the 91 papers retrieved from the databases were selected for this review.
In recent years, there has been a significant surge in research focused on AI, the IoT, and cybersecurity for PV systems driven by advances in technology and a growing need for sustainable energy solutions. This trend is reflected in the increasing number of publications addressing various aspects of PV systems, including energy efficiency, system integration, and the development of smart, autonomous PV systems. Figure 6 illustrates a word cloud for the related research works found on Google Scholar between 2018 and 2023. The number of published articles related to photovoltaic systems has steadily increased, indicating the growing academic and industrial interest in harnessing solar energy. This increase in research activity highlights the potential of PV systems to meet global energy demands and the role of innovation in enhancing their efficiency and integration into modern infrastructures.

### 3.2. Requirements for PV Predictive Maintenance

Predictive maintenance for photovoltaic (PV) systems must be implemented correctly, which depends on a number of important requirements. Among them are reliable systems for acquiring and monitoring data in real time [4,33] to provide high-quality information on a range of system characteristics. Predictive model training requires access to extensive historical datasets, including performance data [6], maintenance logs, and results. In order to guarantee prompt responses to performance deviations from expectations, real-time monitoring and alerts are required. In addition to these requirements, it is noteworthy that predictive maintenance for photovoltaic (PV) systems can utilize alternative approaches like drone inspections employing thermal imaging and deep learning techniques [56]. By enabling early fault detection, performance analysis, and anomaly identification, these techniques offer insightful information about the state of PV panels. Deep learning combined with drone-based thermal inspections further improves predictive maintenance efforts, providing a complete solution for long-term PV system reliability.

### 3.3. Recent Methods and Opportunities for PV Predictive Maintenance

**Figure 7:**
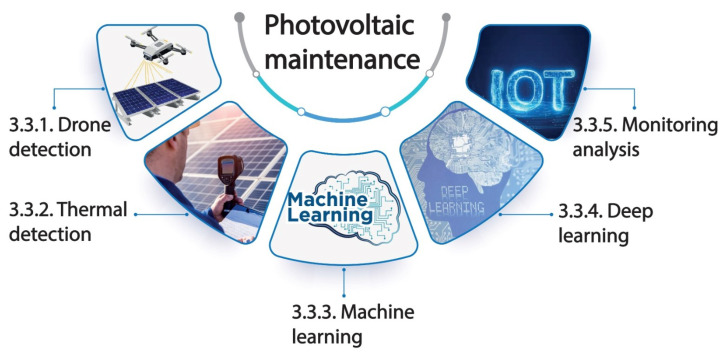
Five recent techniques used for PV PdM.

In recent years, the field of photovoltaic (PV) predictive maintenance has witnessed significant advancements driven by technological innovations and a growing emphasis on sustainable energy production. Figure 7 depicts the recent methods in PV predictive maintenance. All recent papers published in the last five years, as well as the most cited works, are reviewed in detail in Table 1, utilizing the previously mentioned techniques for predictive maintenance in photovoltaics.

#### 3.3.1. Drone Detection

In terms of PV predictive maintenance, drones fitted with thermal imaging cameras have revolutionized the field. The health of PV panels can be quickly and thoroughly evaluated due to these aerial surveys. Temperature variations visible in the thermal images captured from above help identify potential hotspots or defects that may not be obvious to the unaided eye. This technique provides a versatile solution for finding problems early on and scheduling maintenance [57,58].

#### 3.3.2. Thermal Detection

Even in the absence of drone use, thermal inspections are an essential component of photovoltaic (PV) predictive maintenance [59]. During these inspections, infrared pictures of the PV panels are captured using thermal imaging cameras mounted on the ground. Technicians can identify potential problems like hotspots, defective cells, or connections by evaluating the temperature distribution among the panels. Ground-based thermal inspections can be carried out more regularly and are more affordable than aerial drone surveys. Despite not having an aerial perspective, they are still a useful tool for identifying early indications of PV array deterioration or malfunction [60].

#### 3.3.3. Machine Learning

Predictive maintenance has seen an increase in the use of machine learning algorithms. These algorithms are able to forecast possible malfunctions or performance variations by analyzing both historical and current data. Machine learning models can assist in prioritizing maintenance tasks and optimizing inspection and repair schedules by recognizing patterns and trends [61].

#### 3.3.4. Deep Learning

Deep learning, a subset of machine learning, has emerged as a powerful method for predictive maintenance in PV systems. Deep neural networks can analyze vast amounts of historical data, including temperature profiles, energy production, and environmental factors, to predict performance degradation or failures. These models can identify complex patterns and anomalies that may elude traditional methods. By continuously analyzing real-time data, deep learning algorithms can provide early warnings of potential issues, allowing for proactive maintenance and minimizing downtime. The combination of deep learning and predictive maintenance strategies has the potential to revolutionize the way PV systems are managed, ensuring higher reliability and more efficient energy production [62,63].

#### 3.3.5. Monitoring Analysis

The monitoring system handles data acquisition, real-time monitoring, and control of the power plant. It provides critical insights into process behavior, instrumentation status, controller integrity, and alarms, all accessible through operation consoles. The system ensures compliance with national and international grid codes, enabling grid-compatible feed-in at high-voltage levels. A human–machine interface (HMI) visualizes real-time measurements and supports on-site operation management [64]. High availability is achieved through redundant and fault-tolerant configurations for key components, ensuring reliability and minimizing downtime. The system leverages modern technologies, including TCP/IP and the IoT, for enhanced flexibility and performance [65]. The ability to monitor PV systems in real time due to the Internet of Things (IoT) has completely transformed maintenance procedures [66]. PV array sensors constantly gather data on a range of parameters, such as voltage, current, and temperature. Real-time analysis is enabled by transmitting these data to a central platform [67]. The instantaneous detection of anomalies allows for the prompt initiation of maintenance measures, which minimizes downtime and maximizes energy production [68,69,70].

The Ethernet protocol operates at the data link layer of the TCP/IP networking model and uses a system known as Carrier Sense Multiple Access with Collision Detection (CSMA/CD). This system ensures that multiple devices on the same network can share the transmission medium, typically copper or fiber-optic cables, and avoid data collisions. In this study, we developed a Python script to read data from the SMA inverter with the Modbus Protocol. On the other hand, Wi-Fi allows wireless network connections. It connects devices to the LAN or the Internet. It uses radio waves to transmit data between devices and routers. In our application, Wi-Fi can send data from the edge device to the cloud database.

**Table 1:** Review of five recent five methods for PV PdM.

Paper	Year/Citation	Summary of Paper	Method				
			D.D	T.D	M.L	D.L	M.A
[71]	2020/60	The system automates faulty PV module detection with drone assistance and incorporates thermal image processing for enhanced fault detection. Validated in a real 1 MW PV power station.	✓				
[59]	2020/11	Synchronized thermography (ST) and transient thermography (TRT) yielded equally accurate results for outdoor IR imaging, facilitating on-site defect identification in PV power plants for maintenance staff.		✓			
[72]	2020/117	Through extensive experiments comparing the proposed model to established models like VGG16, ResNet50, Inception V3, and MobileNet, the proposed deep learning solution proved its capacity for accurate defect detection in electroluminescence images.				✓	
[73]	2022/17	The CNN-based method surpassed alternative machine learning approaches, including k-nearest neighbor, random forest, decision tree, and support vector machine, for the specific problem at hand.				✓	
[74]	2021/24	The method combines IR cameras for remote temperature capture and a deep neural network, employing a residual network structure and ensemble technique, for precise detection and classification of anomalous solar modules based on IR images.		✓		✓	
[75]	2020/90	The study uses isolated deep learning based on the CNN algorithm to automatically detect defects in photovoltaic modules in infrared images.		✓		✓	
[76]	2022/05	The paper presents an embedded system that uses deep conventional neural networks and infrared thermography images to diagnose and detect faults in photovoltaic (PV) modules.		✓		✓	
[77]	2018/24	The study explores drone intelligence and automation, emphasizing recognition technologies (RTs), artificial intelligence (AI), and machine learning (ML) as key components.	✓		✓	✓	
[49]	2018/91	The paper explores a predictive maintenance approach for photovoltaic (PV) systems that employs an anomaly detection algorithm based on comparing measured and predicted AC power production, using the ANN algorithm.	✓		✓	✓	
[78]	2020/17	The paper presents a feature point matching method with homography translation, derivation of temperature data, and normal/abnormal decision techniques, leveraging drone technology and thermal imaging.	✓	✓			
[79]	2022/10	A novel PV panel condition monitoring and fault diagnosis method is introduced that employs a U-Net neural network for image segmentation and leverages contour features in true-color infrared images for analysis, greatly enhancing processing efficiency.		✓		✓	
[80]	2022/04	An ensemble-based deep neural network (DNN) model is introduced for PV module failure identification, which utilizes a dataset containing both faulty and healthy modules, focusing on feature extraction and fusion to improve classification improvement, with performance compared to existing techniques.				✓	
[81]	2019/38	The paper presents a machine learning model that utilizes an ANN to predict PV panel output under changing conditions and introduces a monitoring system designed to handle heterogeneous panels and enable online data logging, notifications, updates, and data analysis.			✓		✓
[82]	2022/15	The paper presents an Intelligent Monitoring System (IMS) for photovoltaic systems using cost-effective hardware and the IoT, which features a personal cloud server for data storage and web monitoring, as well as deep ensemble models for fault detection and power prediction, offering versatility and efficiency for diverse PV power plant applications.				✓	✓
[83]	2020/20	The paper proposes a novel fault diagnostic scheme in two stages: feature extraction through current-voltage (I-V) analysis and optimization using a genetic algorithm (GA) for parameter tuning in a support vector machine (SVM) classifier, along with feature selection, to achieve higher performance for diagnosing faults in PV systems.			✓		✓
[49]	2018/88	The paper presents a predictive maintenance solution for PV systems, using an anomaly detection algorithm based on AC power production comparisons. It employs an artificial neural network (ANN) model trained with solar irradiance and panel temperature data, allowing for effective maintenance planning tailored to each plant.			✓		✓
[63]	2018/23	The paper introduces a method for monitoring photovoltaic panel performance using convolutional neural networks (CNN) to predict daily electrical power curves by analyzing neighboring panels. Significant deviations between predicted and observed power curves can serve as indicators of panel malfunctions, facilitating efficient maintenance.				✓	✓
[84]	2022/06	As an appropriate option for predictive maintenance, ANNs are recommended in the study to predict solar irradiance and ambient temperature because of their learning capabilities and robustness.			✓		
[57]	2020/06	The study evaluates a recently built solar photovoltaic system situated on the roof of a residential building using an online monitoring platform accessible via the Internet. The results are useful for predicting performance.					✓
[85]	2020/35	The purpose of this paper is to present an open-source, affordable Internet-of-Things solution that can intelligently gather data and continuously monitor the environmental conditions and power produced by solar power plants.					✓

#### 3.3.6. Comparative Cost Analysis of Predictive Maintenance Methods

For a photovoltaic system, choosing the right predictive maintenance technique is crucial and heavily influenced by cost. Inspections using drones are typically more costly, particularly when large-scale installations are involved. Although they are less expensive, ground-based thermal inspections might not be as thorough as aerial surveys. IoT monitoring entails one-time setup fees in addition to continuous data management costs. Robust datasets and the expertise necessary to create and apply these models are prerequisites for machine learning and deep learning. Selecting the best approach entails balancing these cost considerations against the particular requirements and PV system size, with the ultimate goal of striking the best possible balance between cost, dependability, and efficiency. Table 2 provides a broad cost comparison. However, it is crucial to recognize that these expenses may fluctuate over time and exhibit variations based on specific circumstances.

## 4. Results and Discussion

With five well-known techniques at the forefront, the field of predictive maintenance in photovoltaics has advanced significantly. Although drone-based thermal inspections provide thorough aerial views, they are subject to weather-related delays and high costs. While less expensive than other options, ground-based thermal inspections do not offer the same comprehensive view. Although implementation complexity and data security are important factors to take into account, the IoT and real-time monitoring are reliable tools for early anomaly detection. While machine learning is very good at identifying patterns, it needs high-quality data and experience. Deep learning has become a potent technique as well, employing neural networks to analyze large datasets for accurate performance optimization and early fault detection. When combined, these techniques highlight how dedicated the PV system industry is to sustainability, dependability, and efficiency.

The research questions described in the literature review planning protocol allowed us to compare the five proposed methods for PdM related to solar systems, and the advantages and disadvantages of each technique are reported in Table 3.

The best predictive maintenance performance can be achieved by combining multiple methods, especially monitoring and machine learning on an edge device. This is because each method has its own strengths and weaknesses, and by combining them, we can overcome the limitations of each individual method.

In fact, monitoring can be used to collect data from a variety of sensors (current, voltage, irradiance, and temperature sensors), while machine learning can be used to analyze these data and identify patterns that may indicate problems. By combining these two methods, we can predict when an asset is likely to fail and obtain a more accurate picture of its current state.

### 4.1. Challenges in Predictive Maintenance Implementation

The adoption of predictive maintenance in the renewable energy sector presents several challenges that impact the efficiency and reliability of solar panel systems. These challenges arise from both technical and operational constraints and are summarized below.

#### 4.1.1. Model Complexity

Developing models that achieve a balance between computational complexity and prediction accuracy remains a significant challenge. Overly complex models may yield higher accuracy but are computationally expensive and less practical for real-time applications. In contrast, simpler models may lack the precision needed for accurate maintenance predictions.

#### 4.1.2. Data Quality and Availability

Reliable predictive maintenance relies heavily on high-quality data collected over time. However, data inconsistencies, sensor malfunctions, or limited historical data can hinder model development and validation.

#### 4.1.3. Operational Constraints

The integration of predictive maintenance strategies into existing operational frameworks may face challenges such as resistance to new technologies, the cost of upgrading equipment, or the need for specialized expertise.

#### 4.1.4. Potential Solutions and Mitigation Measures

To address these challenges, the following strategies can be considered:

- **Pretrained Models with Hyperparameter Optimization:** Leveraging pretrained models and fine-tuning their hyperparameters can enhance both accuracy and efficiency, reducing the time required for training and deployment.
- **Adaptive Algorithms:** Implementing algorithms that adapt to changing environmental conditions in real time can enhance model robustness and reduce uncertainties.
- **Data Preprocessing and Augmentation:** Employing techniques to preprocess data and simulate additional scenarios can compensate for missing or inconsistent data.

By tackling these challenges, the renewable energy industry can enhance the reliability and efficiency of solar panel systems, ensuring sustainable operations and improved energy output.

### 4.2. Best Practices for Cybersecurity in Solar Plants

When implementing a cybersecurity program for a solar plant, a holistic, defense-in-depth strategy combining digital and physical security measures is essential. The following key practices are recommended:

- **Conduct Regular Cyber Risk Assessments:** Perform walkthroughs, Hazard and Operability (HAZOP) reviews, failure modes and effects analysis (FMEA), and other assessments to identify vulnerabilities and gaps.
- **Implement Robust Security Protocols:** Use firewalls, intrusion detection systems, multi-factor authentication, role-based access control, periodic audits, and proactive system updates to secure infrastructure.
- **Implement Strong Authentication and Access Controls:** Strong authentication mechanisms, such as mandatory multi-factor authentication (MFA), are crucial for securing system access points. Role-based access controls further minimize unauthorized access by limiting access to sensitive systems based on job functions. Regular audits ensure compliance with these policies and help identify vulnerabilities.
- **Regular Software Updates and Patch Management:** Outdated software poses significant risks, as attackers can exploit known vulnerabilities. A robust patch management system ensures timely updates and security patches for all devices and software. Automated systems streamline the update process, reducing disruption while addressing emerging threats.
- **Network Segmentation:** IT/OT convergence enhances manufacturing by improving efficiency, supply chain management, and sustainability. It enables real-time data use for predictive maintenance, resource optimization, and closed-loop quality control. Integrating factory data into IT systems supports better decision making and digital twins for efficiency. Network segmentation further boosts security by isolating IT and OT systems, minimizing risks, and containing threats.
- **Real-Time Monitoring and Intrusion Detection Systems (IDSs):** Real-time monitoring and IDSs detect suspicious activities and breaches in real time, enabling swift responses. These systems flag unauthorized access and identify deviations from normal behavior, providing an extra layer of security and facilitating proactive threat detection.
- **Backup and Disaster Recovery Plans:** Regularly backing up critical data and storing it securely offline minimizes data loss during incidents. Disaster recovery plans outline steps for restoring operations and should be tested frequently to ensure readiness for potential cyberattacks.
- **Enhance Workforce Awareness:** Provide cybersecurity and physical security training during onboarding, teach employees to recognize phishing attempts, create strong passwords, and protect sensitive data.
- **Develop Incident Response Plans:** Establish and practice predefined plans to effectively respond to cybersecurity incidents.
- **Engage External Cybersecurity Experts:** Use experts for vulnerability scans, red teaming, and threat assessments to identify and mitigate risks.

A cybersecurity program must remain active and adaptive to address emerging threats and vulnerabilities. Continuous improvement through regular updates to protocols, training, and risk assessments is crucial to safeguard sensitive data and infrastructure. Failure to adapt may expose solar plants to costly security breaches.

### 4.3. Emerging Trends and Open Research Issues in Cybersecurity for Solar Plants

Emerging trends and open research issues in cybersecurity for solar plants require a comprehensive approach to address both current challenges and future advancements. This subsection reviews key trends in the field, including the application of artificial intelligence for threat detection, the adoption of secure network protocols like TLS, and the integration of the IoT and edge computing. These trends not only enhance the security of solar plant systems but also pave the way for future research directions in safeguarding solar energy infrastructure.

#### 4.3.1. Artificial Intelligence for Cybersecurity

Securing solar plants requires a comprehensive approach that addresses both cyber and physical threats. The integration of AI and ML technologies has proven essential for detecting and preventing cyber threats. These advances are revolutionizing the way we secure solar plants, offering numerous benefits [86].

#### 4.3.2. Secure Network Protocols

Another important trend involves the adoption of secure network protocols, such as Transport Layer Security (TLS) or similar approaches, to protect data transmission in solar plant networks. These protocols ensure the integrity and confidentiality of data, mitigating the risk of cyberattacks on operational systems [87].

#### 4.3.3. Integration with IoT and Edge Computing

The integration of cybersecurity measures with Internet of Things (IoT) devices and edge computing is another emerging area. As solar facilities increasingly rely on IoT devices for monitoring and control, securing these devices and the communication between them becomes a critical research issue [88].

#### 4.3.4. Blockchain Technology

The integration of blockchain technology into PV plants enhances security, ensures traceability, and improves the efficiency of energy transactions. By providing a decentralized and immutable ledger, blockchain significantly strengthens the security of PV plants, thereby reducing the risks associated with centralized control, such as single points of failure and exposure to cyberattacks [89].

## 5. Conclusions

The current state of predictive maintenance techniques for photovoltaic (PV) systems has been thoroughly examined in this review paper. This study investigates five widely used techniques—drone-based thermal inspections, ground-based thermal inspections, IoT monitoring, machine learning, and deep learning— highlighting various approaches to enhancing the reliability and efficiency of PV systems.

The significance of selecting the appropriate technique depends on the available data, financial constraints, and the need for real-time monitoring. Ground-based inspections offer a cost-effective solution for routine maintenance, while drone inspections, although providing unmatched aerial views, are limited by weather conditions and higher costs. IoT monitoring stands out as an effective tool for early anomaly detection; however, its successful implementation requires addressing significant cybersecurity challenges, including securing communication protocols, protecting sensitive data, and safeguarding against cyberattacks. Machine learning and deep learning techniques are pivotal in fault detection and system optimization. While deep learning offers high accuracy, machine learning excels at interpreting patterns, although both require high-quality, specialized data for best results.

An essential aspect of integrating predictive maintenance techniques is cybersecurity, especially when utilizing IoT and machine learning techniques. As PV systems become more connected and data-driven, they are increasingly vulnerable to cyberattacks, such as data injection and denial-of-service attacks. Ensuring the security of IoT devices, securing communication networks, and implementing strong authentication and access controls are critical to protecting the integrity and operation of PV systems. This highlights the importance of incorporating cybersecurity measures into the development of monitoring systems to prevent malicious interference and ensure the safety of the data being collected.

In our forthcoming work, we will focus on creating affordable and secure monitoring systems, as well as seamlessly integrating machine learning algorithms, particularly artificial neural networks (ANNs), which have been identified as one of the most effective approaches in the literature. By developing solutions that are both cost-effective and secure, we aim to enhance the sustainability, reliability, and efficiency of solar energy production while addressing the growing cybersecurity risks associated with the increasing digitalization of PV systems. 

## Figures and Tables

**Table 2:** Cost analysis of predictive maintenance methods.

Method	Cost (USD)	Detail
Drone-based thermal inspections	1500–2000	Drone camera and software
Ground-based thermal inspections	500–800	Camera
IoT monitoring	50–100	Data logger and software
Machine learning	Open-source	ANN algorithm
Deep learning	Open-source	CNN algorithm

**Table 3:** Comparison of PdM for the five techniques.

Method	Advantage	Disadvantage
Drone	Versatile	Expensive
Thermal camera	Detects overheating components	Depends on environmental factors
Monitoring	Simple to implement	Analyzing data manually
Machine learning	Automatic failure detection	Significant labeled data to train
Deep learning	Complex patterns in data	Significant labeled data to train

## Data Availability

This article uses private data that are made available through a GitHub repository. The dataset and code used for the machine learning comparison in this study can be accessed at the following link: https://github.com/Ledmaoui/Solar-Panel-Anomalies-Detecting (accessed on 1 January 2024).

## Abbreviations

The following abbreviations are used in this manuscript:

ANNs	Artificial neural networks
CNN	Convolutional neural network
ECE	External Cybersecurity Experts
AI	Artificial intelligence
IoT	Internet of Things
IT/OT	Information Technology/Operational Technology
LSTM	Long Short-Term Memory
AUC-ROC	Area Under the Receiver Operating Characteristic Curve
CC BY	Creative Commons Attribution
ML	Machine learning
PdM	Predictive maintenance
RF	Random forest
SVM	Support Vector Machine
SCADA	Supervisory Control And Data Acquisition
SVR	Support Vector Regression
MFA	Multi-Factor Authentication
HAZOP	Hazard and Operability
FMEA	Failure modes and effects analysis
DL	Deep learning
IDS	Intrusion Detection Systems
OT	Operational Technology
TCP-IP	Transmission Control Protocol/Internet Protocol
TLS	Transport Layer Security
WI-FI	Wireless Fidelity
